# Understanding Socioeconomic and Psychological Vulnerabilities in Post-Disaster Recovery: Insights from the Displaced New Orleans Residents Survey

**DOI:** 10.3390/ijerph23030368

**Published:** 2026-03-13

**Authors:** Tanjila Rashid Rhythy, Yian Xu, Da Hu

**Affiliations:** 1 Department of Civil and Environmental Engineering, Kennesaw State University, Marietta, GA 30060, USA; trhythy@students.kennesaw.edu; 2 Department of Psychological Science, Kennesaw State University, Kennesaw, GA 30144, USA; yxu11@kennesaw.edu

**Keywords:** Hurricane Katrina, socioeconomic factors, K6 mental illness scale, post-traumatic stress disorder checklist (PCL), depression, perceived stress scale (PSS), regression analyses

## Abstract

**Highlights:**

**Public health relevance—How does this work relate to a public health issue?**

- Examines long-term socioeconomic and psychological vulnerabilities among displaced residents following Hurricane Katrina, a major public health disaster.
- Integrates mental health outcomes (PTSD, depression, perceived stress, psychological distress) with socioeconomic and housing factors relevant to disaster recovery.

**Public health significance—Why is this work of significance to public health?**

- Identifies disproportionately affected populations based on race, income, education, insurance status, and household characteristics.
- Demonstrates how structural damage and socioeconomic insecurity independently and jointly shape post-disaster mental health outcomes.

**Public health implications—What are the key implications or messages for practitioners, policy makers and/or researchers in public health?**

- Highlights the critical role of insurance coverage, education, and income stability in mitigating post-disaster psychological distress.
- Supports the need for targeted, equity-focused disaster recovery and mental health interventions to improve long-term community resilience.

**Abstract:**

Communities susceptible to disasters frequently endure severe socio-economic and psychological repercussions. Therefore, it is essential to thoroughly understand the various vulnerabilities encountered by different groups. Residents of New Orleans, Louisiana, faced significant hardships after Hurricane Katrina hit on 29 August 2005. A multitude of individuals lost their residences, while others, regrettably, lost family members. The Displaced New Orleans Residents Survey (DNORS) offered significant insights into individuals and households living in New Orleans immediately prior to Hurricane Katrina’s impact in August 2005. The survey interview was conducted from mid-2009 until mid-2010. This study utilizes DNORS public data files to evaluate socio-demographic characteristics pertinent to the analysis, including age, gender, race/ethnicity, marital status, household income, education level, employment status in 2005, and insurance coverage, alongside psychological measures such as mental health symptoms, posttraumatic stress, depression, and perceived stress. The research employs various regression techniques to identify the at-risk categories affected psychologically and physically by the hurricane. These findings may aid policymakers in developing targeted post-disaster recovery strategies, thereby promoting more resilient and sustainable communities.

## 1. Introduction

Hurricane Katrina, classified as a Category 3 hurricane, impacted the Gulf Coast on 29 August 2005, representing one of the most catastrophic natural catastrophes in the history of the United States [1,2,3]. The hurricane caused estimated property damages between $70 billion and $125 billion and resulted in over 1400 fatalities, marking an unparalleled level of destruction in human casualties and economic consequences [1]. The city of New Orleans, Louisiana, suffered severe devastation, resulting in substantial damage to infrastructure, considerable fatalities, widespread displacement of inhabitants, and lasting economic consequences. Following the event, New Orleans encountered nearly maximal flooding, leading federal entities, including the Federal Emergency Management Agency (FEMA), to utilize remote sensing imagery for evaluating the breadth and depth of the inundation [4]. Approximately 71% of inhabited housing units incurred damage, rendering it the most substantial residential catastrophe in U.S. history [5]. Approximately 183,000 residential units were significantly damaged or obliterated, requiring a near-complete evacuation of the city and distributing inhabitants nationwide [1,2]. Approximately 90,000 square miles of the Gulf Coast were impacted, resulting in the evacuation of 400,000 people and economic losses estimated at $200 billion [3].

The existing body of literature extensively documents the profound and multifaceted mental health consequences that follow in the wake of natural disasters. Consistently, research highlights elevated rates of psychological distress, encompassing conditions such as posttraumatic stress disorder (PTSD), depression, anxiety disorders, and substance use disorders among affected populations [6]. These conditions not only represent immediate responses to trauma but can also manifest as long-term psychological sequelae, significantly impacting the lives of disaster survivors [7]. Studies focusing on the aftermath of Hurricane Katrina, for instance, have revealed robust associations between disaster-related experiences—including exposure to the storm, displacement, and loss and subsequent psychological distress, underscoring the urgent need for effective mental health support and intervention strategies in disaster-affected communities [6].

## 2. Review of Relevant Literature

### 2.1. Methodological Considerations in Disaster Research

Research into the psychological consequences of disasters, particularly in the long term, faces several pervasive methodological challenges relating to sampling, study design, and the measurement and conceptualization of outcomes [8,9].

One of the most crucial methodological considerations is the sampling procedure [10,11]. Many existing disaster studies have been compromised by a failure to use random or probability sampling techniques, which limits the interpretation and generalization of psychopathology prevalence rates [8,9,12]. Only 39% of disaster studies utilized some form of random or representative sampling [3]. Some studies, such as those focusing on Hurricane Katrina survivors, relied on convenience samples, meaning results cannot be assumed generalizable to all individuals exposed to a natural disaster [13,14].

Research conducted in post-disaster settings faces unique challenges, such as low initial response rates, partly because it is difficult to distinguish eligible households from vacant or abandoned ones [15]. The high transience and fluid nature of displaced populations also complicate follow-up efforts, introducing potential bias concerning who can be located and re-interviewed [15,16]. Furthermore, using methods such as phone-based, self-report designs may introduce misclassification errors regarding race and other risk factors [17].

A significant limitation in the extant literature is the lack of longitudinal data spanning the period before and after the disaster [14,18]. Without pre-disaster data on mental health status, researchers cannot isolate the effects of the disaster from pre-existing conditions, potentially overestimating the impact of disaster-related exposures [19]. A study found that pre-disaster mental health functioning is one of the strongest predictors of post-disaster mental health outcomes, reinforcing the necessity of baseline data [14].

Relatively few longitudinal studies track the course of mental health problems over prolonged periods, with most US disaster studies being cross-sectional and conducted within the first year post-disaster [18,20]. Long-term studies, tracking survivors for a decade or more, are vital for understanding the enduring consequences and recovery process, particularly after mega-disasters like Hurricane Katrina, where full recovery can take years or decades [14,21]. Cross-sectional designs inherently prevent researchers from drawing conclusions about causal mechanisms or studying recovery factors over time [6,8,17].

There is a recognized need to advance the conceptual rigor of disaster research, moving beyond purely descriptive studies [9]. Researchers must conceptually and empirically separate socioeconomic circumstances from traumatic circumstances in disaster-exposed samples [13]. Furthermore, many disaster studies traditionally focused narrowly on post-traumatic stress disorder (PTSD) and related psychological symptoms [10]. There is a call for research to assess the impact across broader psychosocial domains, including symptoms, social support, and perceived discrimination, to gain a more comprehensive understanding of disaster reactions [22]. Methodological shortcomings also include the failure to routinely analyze outcome data while controlling for potential demographic moderators, such as gender and age, or adequately distinguishing between clinical and nonclinical samples [3,9].

### 2.2. Factors Influencing Mental Health After Hurricane Katrina

A multitude of individual- and community-level factors influence post-disaster mental health, particularly highlighting the increased vulnerability of socioeconomically disadvantaged and demographically specific subgroups [14]. Socioeconomic inequality contributes significantly to post-disaster vulnerability [19,23]. Low-income individuals, such as low-income African Americans following Hurricane Katrina, disproportionately experienced the greatest suffering [6].

Acute socioeconomic decline, characterized by sudden job loss and deficits in access to SES resources following a disaster, can create long-term health disparities independent of stable pre-hurricane SES levels and traumatic experiences [13]. Acute difficulties accessing SES resources were associated with PTSD and chronic pain 4–5 years post-hurricane [10,13].

In African American adults 4–5 years after Hurricane Katrina, those who became unemployed immediately after the hurricane had five times higher odds of experiencing a cardiometabolic event, three times higher odds of meeting criteria for Major Depressive Disorder (MDD), and two times higher odds of experiencing chronic pain compared to those who maintained employment [3,6,24]. Financial strain following Katrina among Black survivors was associated with a higher level of PTSD symptoms [19].

Income levels showed a consistent protective effect. Following Hurricane Katrina, the negative impact on mental health (number of poor mental health days) was larger among lower income respondents, with consequences decreasing monotonically as income levels increased [21]. In a sample of African American evacuees, income demonstrated the strongest significant effect on psychological resilience (perceived sense of recovery) among all sociodemographic variables examined [6]. Additionally, higher earnings before Hurricane Katrina were found to be protective against developing Post-Traumatic Stress Symptoms (PTSS) alone at the second follow-up (43–54 months post-Katrina) [18].

Ethnic minorities, particularly African Americans, have often been found to face poorer psychological outcomes post-disaster [14,17]. African Americans were found to be at higher risk for post-hurricane mental health difficulties [13]. In one cohort of low-income mothers, Black respondents had a statistically significant 3.62 times greater risk of having co-occurring PTSS and psychological distress (PD) compared to non-Black respondents at the first follow-up (7–19 months post-Katrina), controlling for trauma and pre-existing PD [14]. In Hurricane Sandy survivors, Non-Hispanic Black race was a significant individual-level predictor of higher Posttraumatic Stress (PTS) [24]. In a study conducted after Katrina, the odds for depression were 86% higher for African Americans than Caucasians in univariate analysis. While this difference became non-significant after controlling for sociodemographic factors, preexisting vulnerabilities, social support, and trauma-specific factors, a non-significant trend effect suggested race still played an important role [17].

Women are widely identified as a population subgroup at particular risk for mental health problems after natural disasters [6,14,18,22]. Women experienced a larger negative impact on mental health (poor mental health days) from Hurricane Katrina compared to men [21]. Black female survivors reported more PTSD symptoms and worse mental health outcomes compared to Black males after Katrina [19]. The overall number of female victims in disaster study samples has been shown to be directly related to the magnitude of the psychopathology effect-size estimate [9].

Age also influences vulnerability, though findings can be mixed [21,24]. Younger adults’ mental health was more severely affected by Hurricane Katrina compared to their older counterparts [8,21]. Older adults may possess more resources and life experience for coping [25]. Children and adolescents are specifically noted to be at greater risk for PTSD [6]. In contrast, older age was a significant predictor of higher PTS in a study of Hurricane Sandy survivors [24]. Older women were also more likely to experience PTSS alone or with PD after Katrina [18].

The role of children is complex within studies focused on parents. Nearly two years after Katrina, having a greater number of children in a household was a strong predictor of poor mental health [15]. However, in one study of low-income mothers, the presence of young children (0–5 years) and teenagers (12–17 years) reduced the risk of psychological distress (PD) at the first follow-up [11]. It is hypothesized that adolescents may be more self-sufficient and capable of providing social support during the crisis [18].

Home Insurance, Dwelling Type, and Home Damage: Housing characteristics and loss are critical predictors of mental health outcomes [16]. Home destruction or severe damage was a major covariate of mental illness one year post-Katrina [16]. Home damage was consistently associated with PTSS alone or co-occurring with PD in the short-to-middle term (up to 54 months post-Katrina) [18]. For victims who lost their homes in the 2011 Great East Japan Earthquake, housing recovery was the main determinant of their subjective sense of recovery; whereas well-being (physical/mental health, social ties) was the primary determinant for those who did not lose housing [25].

Structural or economic factors like dwelling type (e.g., living in a trailer) or losing a salaried wage earner were not independently associated with poorer mental health nearly two years post-Katrina, as their effects were largely mediated by social factors and attitudes [15]. The variable of being insured had a significant effect on psychological distress among African American evacuees, suggesting its importance as an economic resource [6,20].

### 2.3. Limitations of Existing Research

Despite its valuable insights, current disaster research has some shortcomings that merit attention. A prevalent issue is the dependence on self-report measures, which can introduce biases including social desirability and recall inaccuracies. Survivors may either underreport or exaggerate their psychological discomfort due to stigma, memory distortions, or the impact of their current emotional states [14]. These constraints need the use of many data sources, including clinical evaluations and longitudinal monitoring, to enhance the accuracy of results.

Furthermore, several studies utilize cross-sectional study strategies, which constrain the capacity to determine causal links between catastrophe exposure and mental health outcomes [10]. In the absence of longitudinal data, distinguishing between pre-existing psychological vulnerabilities and distress generated by disaster remains challenging. Future research should emphasize long-term follow-up evaluations to monitor alterations in mental health over time and enhance comprehension of the recovery trajectory.

A significant concern is the absence of representative samples in catastrophe research. Numerous studies concentrate on certain subpopulations, such as individuals pursuing mental health treatment or those residing in temporary shelters, which may not adequately represent the wider impacted population [26]. Consequently, the generalizability of the findings is frequently undermined. Research utilizing random or probability sampling methods can offer a more thorough understanding of the mental health effects of disasters across various demographic groups.

Cultural and contextual aspects are often neglected in catastrophe mental health research. Individuals’ experiences, expressions, and coping mechanisms for distress might differ markedly across cultural contexts, influencing both symptom manifestation and treatment-seeking behaviors [25]. Standardized diagnostic criteria may not consistently apply to varied populations, resulting in possible underestimations or overestimations of psychological distress. Future research must include culturally appropriate assessment instruments and qualitative approaches to elucidate the complexities of mental health in communities impacted by disasters.

Inconsistencies in findings related to PTSD, acute stress disorder (ASD), emotional distress, and other psychosocial dysfunctions may emerge from variations in methodological approaches, assessment instruments, and diagnostic criteria across diverse studies [25,27]. Standardizing research methodology and integrating multifaceted assessment procedures might alleviate these inconsistencies and enhance the credibility of conclusions derived from disaster mental health research.

Despite the valuable insights offered by existing disaster research, the methodological literature reviewed above largely overlooks the field of disaster epidemiology, which has developed systematic and well-validated frameworks specifically designed for post-disaster data collection. Disaster epidemiology provides structured methodologies, including rapid needs assessments, community-based participatory sampling, and multistage cluster sampling designs, that directly address the challenges inherent in studying displaced and dispersed populations, such as incomplete sampling frames, high residential transience, and difficulty locating eligible households [28]. These approaches have been widely applied in the aftermath of hurricanes, earthquakes, and other large-scale disasters to generate population-representative estimates of health outcomes, including mental health sequelae [29]. The DNORS dataset employed in the present study draws on a probability-based sampling strategy informed by this tradition, which strengthens the generalizability of our findings relative to studies relying on convenience samples. Nevertheless, future research on post-disaster mental health would benefit from more deliberate integration of disaster epidemiology best practices, particularly longitudinal surveillance designs and multi-wave probability sampling to better capture recovery trajectories and reduce the selection biases that continue to limit the field.

### 2.4. Contributions of This Study

This research presents significant contributions to disaster psychology and socio-economic vulnerability assessment. It employs an extensive dataset obtained from the DNORS public data files, which includes a varied population of displaced individuals from New Orleans. The dataset comprises an extensive array of socio-demographic variables, including age, gender, race/ethnicity, home ownership, dwelling type, household income, insurance status, and employment history, in conjunction with essential psychological metrics such as the K6 mental illness scale, PTSD checklist (PCL), depression assessments, and perceived stress scale (PSS). The incorporation of these many elements for a comprehensive examination of the psychological consequences of Hurricane Katrina, yielding profound insights into the resilience and vulnerability of impacted communities.

Secondly, this study presents a comparative analysis of the influence of socio-demographic characteristics on house damage levels and psychological distress among inhabitants of New Orleans. This research examines the relationship between infrastructure loss and mental health effects. Integrating structural damage levels and psychological effects of the affected population, this paper provides a comprehensive perspective on post-disaster recovery.

This study utilizes robust regression techniques to identify critical at-risk populations and furnish suggestions for policymakers. This research identifies the socio-demographic characteristics that most significantly predict psychological suffering and economic instability after a disaster, thereby informing focused intervention measures to better serve vulnerable groups. This study’s findings are crucial for developing disaster response frameworks that incorporate mental health support and economic recovery strategies, thereby fostering more resilient and sustainable communities following catastrophic disasters.

To guide this investigation, the study addresses the following three research questions:

**RQ1.**  *Which socioeconomic and demographic characteristics are associated with the level of structural damage experienced by households during Hurricane Katrina?*

**RQ2.**  *Which socioeconomic and demographic characteristics are associated with psychological distress outcomes including general psychological distress (K6), PTSD symptoms (PCL), depression severity (PHQ-9), and perceived stress (PSS) among displaced New Orleans residents, independent of structural damage?*

**RQ3.**  *How does the inclusion of structural damage as a predictor alter the associations between socioeconomic and demographic characteristics and psychological distress outcomes, and does structural damage independently contribute to mental health outcomes after accounting for background socioeconomic factors?*

## 3. Methodology

### 3.1. Multinomial Logistic Regression

This study employs multinomial logistic regression to examine the associations between socioeconomic and demographic characteristics and the level of structural damage sustained by households during Hurricane Katrina. The dependent variable, damage level, is categorical with four unordered outcomes: “No Damage,” “Damaged but Someone Could Still Live in It,” “Damaged So Badly That Someone Couldn’t Live in It,” and “Destroyed.” Because this outcome does not have a natural numeric ordering that can be assumed to be uniform across categories, and because the predictors include a mix of continuous variables (age, household size) and categorical variables (race, gender, education, income bracket, insurance status, employment status, dwelling type, homeownership, and presence of children), multinomial logistic regression is the appropriate analytical framework. Prior to model estimation, we tested the proportional odds assumption using the Brant test to evaluate whether an ordinal logistic regression model would be appropriate. The results indicated violations of proportional odds for several key predictors, including race and insurance status, confirming that the multinomial specification better fits the data. “Destroyed” was selected as the reference category because it represents the most severe damage outcome and provides the most meaningful baseline for comparing households with lesser degrees of damage.

Let Y represent the dependent variable indicating the extent of building damage, which can assume c unordered categories {0, 1, …, c − 1}, with category 0 designated as the reference category. Let X = (x_1_, x_2_, …, x*_p_*) represent a vector of p independent variables [30]. The multinomial logistic regression model calculates the probability of each outcome category (Equation (1)):

(1) $$\left\{\begin{array}{c} P\left(Y=j\left|X\right.\right)=\frac{exp\left(\beta_{j0}+\beta_{j}^{T}X\right)}{1+\sum_{k=1}^{c-1}exp\left(\beta_{k0}+\beta_{k}^{T}X\right)},\;\;\;\mathrm{for}\;j=1,\ldots ,c-1\\ P\left(Y=0\left|X\right.\right)=\frac{1}{1+\sum_{k=1}^{c-1}exp\left(\beta_{k0}+\beta_{k}^{T}X\right)} \end{array}\right.$$

where $\beta_{j0}$ is the intercept term for category j, and $\beta_{j}$ is the vector of regression coefficients for category j. The log-odds (logit) of belonging to category j in relation to the reference category 0 is expressed by Equation (2):

(2) $$\ln\left(\frac{P\left(Y=j\left|X\right.\right)}{P\left(Y=0\left|X\right.\right)}\right)=\beta_{j0}+\beta_{j}^{T}X,\;\;\;for\;j=1,\ldots ,c-1\;$$

The coefficients β*_j_* denote the influence of the independent variables on the log-odds of belonging to category *j* in comparison to the reference category [30]. Exponentiating the coefficients produces the relative risk ratios (RRRs), which reflect the alteration in the relative risk of belonging to category *j* in comparison to the reference category for a one-unit increment in the predictor variable [31]. An RRR exceeding 1 signifies that elevated predictor values correlate with an augmented relative risk of belonging to category *j* in comparison to the reference category, whereas an RRR below 1 denotes a diminished relative risk [32].

### 3.2. Ordinary Least Squares Regression

Ordinary least squares (OLS) regression is a statistical method widely used to model the linear relationship between one or more independent variables and a single continuous dependent variable [33]. The fundamental principle of OLS is the least squares method, which aims to build a line (or hyperplane in the case of multiple independent variables) that best fits the data by minimizing the sum of the squared deviations of each data point from the line [33]. This line serves as the most accurate linear depiction of the spread of the data points [33].

To examine the associations between socioeconomic and demographic characteristics and post-disaster psychological distress, this study employs Ordinary Least Squares (OLS) regression. Four psychological outcomes are modeled separately: general psychological distress (K6 scale, range 0–24), PTSD symptoms (PCL, range 17–85), depression severity (PHQ-9, range 0–27), and perceived stress (PSS, range 0–16). Each outcome is treated as a continuous dependent variable, consistent with standard practice in the disaster mental health literature where validated psychometric scales with sufficient numeric range are routinely analyzed using linear regression methods [34]. The independent variables are the same socioeconomic and demographic predictors used in the damage analysis: age, gender, race/ethnicity, education level, household income bracket, employment status, insurance coverage, dwelling type, homeownership status, household size, and presence of children. In the third analysis, damage level is additionally included as a predictor to examine whether structural loss independently contributes to psychological distress after accounting for background socioeconomic characteristics.

OLS regression coefficients are estimated by minimizing the sum of squares of the differences between the values fitted by the regression plane and the observed values in the data [33]. A multiple linear regression model can be represented as

(3) $$y=\beta_{0}+\beta_{1}x_{1}+\beta_{2}x_{2}+\cdots +\beta_{p}x_{p}+\varepsilon \;$$

where the following definitions are used:

y is the dependent variable;

x_1_, x_2_, …, x_p_ are the independent variables;

β_0_ is the intercept;

β_1_, β_2_, …, β_p_ are the regression coefficients representing the linear effect of each independent variable on the dependent variable;

ε is the error term, representing the unexplained variance in the dependent variable [35].

It is crucial to note that OLS regression relies on several data assumptions that a researcher must check before conducting the analysis [33]. Violations of assumptions, such as non-constant error variance often associated with a skewed dependent variable, may necessitate transformations of the dependent variable, such as a logarithmic transformation, to meet the model’s requirements [35]. To facilitate comparisons between independent variables measured in different units, standardized regression coefficients (β or Beta weights) are often calculated [33]. These coefficients are derived by standardizing all unstandardized regression coefficients based on the ratio of the standard deviation of the independent variable to the standard deviation of the dependent variable, allowing for a direct comparison of their relative effects within the model [33].

## 4. Data Description

The Displaced New Orleans Residents Survey (DNORS) was executed five years post-Hurricane Katrina to evaluate its social, demographic, health, and economic effects on former residents of Orleans Parish [36]. The survey encompassed individuals who stayed in New Orleans and those who relocated, collecting data by nationwide telephone and in-person interviews. This study employs DNORS data to analyze the impact of socioeconomic and demographic factors on psychological outcomes and the extent of structural damage.

Table 1 provides the socio-demographic information of the residents. The dependent variable in this study is the level of damage reported by respondents and it is categorized into four levels: “no damage,” “damaged but someone could still live in it,” “damaged so badly that someone couldn’t live in it,” and “destroyed.” Relevant variables that were collected from the DNORS database include race, income, employment, education, dwelling type, insurance, and homeownership along with psychological data such as K6 mental illness scale, PTSD, PHQ-9 Depression, and perceived stress scale. Out of the initial 1761 households surveyed, 1753 provided information on their level of damage. However, due to missing data on some variables, households with incomplete information were excluded from the analysis. After data cleaning, a total of 1682 households remained for the regression analysis of the damage levels.

Table 2 provides the psychological variables utilized in this study. Out of the initial 1761 households surveyed, a total of 1629 provided all of the information required for the psychological analysis of the residents. Due to missing data on some variables, households with incomplete information were excluded from the analysis. After data cleaning, a total of 1629 households remained for the study.

There are four types of psychological data obtained from DNORS dataset: Mental Illness Scale (K6 Scale), Post-Traumatic Stress Disorder (PCL), Depression (PHQ9), and Perceived Stress (PSS). The descriptions of these variables are described below:

K6 Mental Illness Scale: The Kessler Psychological Distress Scale (K6) is a brief, 6-item screening tool designed to measure psychological distress in community and primary care settings [38,39]. The questions assess how frequently a person experienced feelings of nervousness, hopelessness, restlessness, depression, effort, and worthlessness over a specific period [38,40]. K6 is a scale of non-specific psychological distress that was used to screen for anxiety and mood disorders of the survivors of the hurricane Katrina in the previous 30 days. For the K6 mental illness scale, the respondents were asked to rate how often they experienced various feelings associated with depression and anxiety over the past 30 days. K6 score was only created if a respondent answered all six questions.

Post-Traumatic Stress Disorder: Post-traumatic Stress Disorder (PTSD) is characterized by intrusive, involuntary cognitive phenomena such as flashbacks and nightmares, alongside increased allocation of attentional resources towards threatening stimuli [41]. It is also associated with concentration problems and memory deficits [41,42]. The PTSD Checklist (PCL) was used in the DNORS to assess post-traumatic stress disorder (PTSD) of the New Orleans residents. For the Post-Traumatic Stress Disorder Checklist (PCL), respondents were asked about psychological problems or complaints that they experienced in the past month because of Hurricane Katrina. The PCL includes 17 questions that correspond to each of the single items comprising the symptoms of PTSD from the Diagnostic and Statistical Manual of Mental Disorders—Fourth Edition (DSM-IV). The PCL in DNORS is focused on respondents’ Katrina-related experiences.

Depression: The Patient Health Questionnaire-9 (PHQ-9) is a widely used, 9-item instrument for screening and measuring the severity of depression in clinical research and practice [43]. PHQ-9 was used to assess major depression of Katrina survivors of New Orleans. The PHQ-9 assesses symptoms and functional impairment in order to determine the likelihood of a respondent has major depression and also assesses the severity of the respondent’s symptoms. The PHQ-9 is based on the diagnostic criteria from the DSM-IV. The nine-item Patient Health Questionnaire (PHQ-9) asks about symptoms associated with significant depression and whether respondents had experienced these symptoms in the past 30 days. These symptoms include feeling down, having trouble sleeping, and having little energy.

Perceived Stress: The Perceived Stress Scale (PSS) is a questionnaire that measures an individual’s perception of stress [44,45]. DNORS used the Perceived Stress Scale (PSS) to assess respondents’ experiences of psychological stress. DNORS used a four-item version of the scale. The PSS items were designed to assess the degree to which respondents found the circumstances in their lives unpredictable, uncontrollable, and overloaded. It provides a general measure of perceived stress rather than the experience resulting from the specific stressor.

By analyzing these psychological indicators alongside socio-demographic factors (e.g., income, race, employment, insurance coverage), the study aims to identify at-risk populations and inform policies for disaster preparedness and mental health support. This data-driven approach ensures that disaster recovery strategies address not only economic losses but also the psychological well-being of affected communities, promoting holistic and sustainable recovery efforts.

## 5. Results

All findings reported below are associative in nature and reflect correlations observed in a cross-sectional dataset with self-reported outcomes. These results are subject to residual confounding from unmeasured variables, and the cross-sectional design does not permit causal inference. Readers should interpret all associations accordingly.

The results are organized into three sequential analyses that together address the three research questions stated in Section 2.4. The first analysis (Section 5.1) addresses RQ1 by examining which socioeconomic and demographic characteristics are associated with differential levels of structural damage across households establishing the landscape of physical vulnerability. The second analysis (Section 5.2) addresses RQ2 by examining the direct associations between those same background characteristics and four psychological distress outcomes, without accounting for damage, isolating the socioeconomic pathways to mental health independent of physical loss. The third analysis (Section 5.3) addresses RQ3 by re-estimating the psychological models with damage level included as an additional predictor, allowing to assess whether structural damage independently contributes to psychological distress and whether the inclusion of damage attenuates the associations observed for socioeconomic variables, which would suggest partial mediation of socioeconomic effects through physical harm. Together, the three analyses provide a layered understanding of the interplay between socioeconomic vulnerability, physical disaster exposure, and post-disaster psychological outcomes.

### 5.1. First Analysis: Regression Analysis of Damage Levels

The multinomial logistic regression analysis was conducted to examine the relationship between various socioeconomic and demographic factors and the levels of building damage experienced by households during Hurricane Katrina. The damage levels were categorized into four outcomes: “No Damage,” “Damaged but Someone Could Still Live in It,” “Damaged So Badly That Someone Couldn’t Live in It,” and “Destroyed.” For this analysis, “Destroyed” was set as the baseline category, allowing for comparisons between households that experienced destruction and those that sustained varying degrees of lesser damage. Table 3 presents the regression results of factors associated with building damage. More detailed results and analysis can be found in our previous study [37].

#### 5.1.1. Comparison Between ‘No Damage’ and ‘Destroyed’

The analysis revealed key predictors when comparing unaffected households to those with destroyed homes. Homeownership is associated with a lower likelihood of no damage versus destruction, with homeowners exhibiting a relative risk ratio (RRR) of 0.33. This suggests that homeowners may have lived in more vulnerable areas or owned older, more susceptible buildings.

Race was important. White households reported no damage more frequently than African American households, with an RRR of 3.93. This highlights potential systemic racial disparities. Insurance coverage was crucial. Households with full insurance coverage reported no damage more frequently (RRR of 3.44), while those with partial coverage reported no damage less frequently (RRR of 0.19) compared to destruction. Age is positively correlated with no damage, with each year increasing the relative risk by 3% (RRR of 1.03). Larger households were less likely to report no damage than destruction, with a relative risk ratio of 0.72.

#### 5.1.2. Comparison Between ‘Damaged but Someone Could Still Live in It’ and ‘Destroyed’

While comparing households with habitable damage to those with destroyed homes, race was a significant factor. White households (RRR of 4.58) and households identified as other races (RRR of 4.55) were more likely to report habitable damage compared to African American households.

Households with children reported less habitable damage than destruction (RRR of 0.65). Prior employment status was significant, as employed individuals were less likely to report habitable damage compared to destruction (RRR of 0.66).

Insurance coverage demonstrated a protective effect. Households with complete loss coverage (RRR of 4.81) and those with significant loss coverage (RRR of 1.84) were more likely to report habitable damage rather than destruction. Household income between $75,000 and $99,999 correlated with a reduced likelihood of reporting habitable damage versus destruction (RRR of 0.28).

#### 5.1.3. Comparison Between ‘Damaged So Badly That Someone Couldn’t Live in It’ and ‘Destroyed’

In comparing households with severe damage making homes uninhabitable to those with destroyed homes, fewer significant variables were identified. Households with children exhibited a protective effect, with an RRR of 0.64 (*p* < 0.05), suggesting they were less likely to face destruction than uninhabitable damage. This may result from factors like living in safer areas or increased investment in housing resilience for family safety.

Insurance coverage was a key predictor. Households with full insurance coverage had an RRR of 2.48 (*p* < 0.05), indicating a higher likelihood of reporting uninhabitable damage rather than destruction compared to uninsured households. This suggests that insurance may have reduced some damage but did not completely prevent it.

Age exhibited a minor but notable impact, with an RRR of 1.01 (*p* < 0.05). Older individuals reported uninhabitable damage more frequently than destruction, though the effect size was minimal. This suggests that older residents’ homes may have been less prone to total destruction, potentially due to superior construction standards or greater experience in disaster preparedness.

### 5.2. Second Analysis: Regression Analysis of Psychological Parameters Without Damage Levels

An ordinary least squares regression analysis was conducted to examine the relationship between various socioeconomic and demographic factors and the psychological parameters experienced by respondents during Hurricane Katrina. The psychological parameters were categorized into four outcomes: “K6 Score,” “PTSD,” “PHQ9 Depression,” and “PSS.” Table 4 presents the regression results of factors associated with psychological outcomes without integrating levels of damage as independent variables.

This analysis, presented in Table 4, aimed to identify which socio-demographic factors were significant in predicting the mental health outcomes of the Displaced New Orleans Residents surveyed after Hurricane Katrina, without considering the direct impact of physical damage to their residences. The psychological outcomes measured were the K6 Mental Illness Scale, PTSD Checklist, PHQ9 Depression, and Perceived Stress Scale. Here is a detailed analysis of each of the outcome variables:

K6 Mental Illness Scale: The K6 Mental Illness Scale assesses psychological distress, with regression analysis revealing key predictors. Age exhibited a significant negative coefficient of −0.04 (*p* < 0.001), indicating that older respondents experienced reduced psychological distress. Gender influenced distress levels, as female respondents reported significantly lower distress than males, with a coefficient of −0.50 (*p* < 0.05).

Race significantly influenced outcomes, with White respondents exhibiting lower general psychological distress (coefficient of −0.67) than the reference group. Children in the household correlated with heightened distress, evidenced by a significant positive coefficient of 0.66 (*p* < 0.05). Higher education levels correlate with reduced psychological distress; those with a high school diploma, some college, or a college degree reported lower distress levels (coefficients of −1.46, −2.06, −2.75, respectively).

Financial security emerged as a protective factor; individuals with fully insured losses reported significantly lower distress (coefficient −1.66, *p* < 0.001). A similar trend was noted for those with most losses covered (coefficient −1.08, *p* < 0.05). Higher household income levels, specifically in the $50,000–$75,000, $75,000–$100,000, and $100,000+ brackets, correlated with reduced general psychological distress, highlighting the link between financial stability and mental well-being.

PTSD Checklist: The PTSD Checklist, assessing symptoms of post-traumatic stress disorder from Hurricane Katrina, identified several significant predictors. Gender differences were apparent, with female respondents indicating lower PTSD symptoms than males (coefficient of −1.96). Consistent with K6 findings, White respondents showed reduced PTSD symptom severity, with a coefficient of −4.07 (*p* < 0.001). Households with children exhibited higher PTSD symptoms (coefficient of 1.74), indicating that children contributed to increased psychological strain post-disaster. Education emerged as a protective factor, with higher educational attainment (high school graduate, some college credit, and college degree) linked to significantly lower PTSD symptoms (coefficients of −2.74, −4.6, −6.08, respectively).

Insurance coverage linked to reduced PTSD symptoms indicates financial stability. Respondents with fully insured losses reported significantly lower PTSD symptoms (coefficient of −5.82). Those with mostly covered losses also exhibited a notable reduction (coefficient of −4.12, *p* < 0.05). Higher household income levels ($20,000–$30,000, $30,000–$50,000, $50,000–$75,000, $75,000–$100,000, and $100,000+) were negatively correlated with PTSD symptoms, reinforcing the link between financial security and psychological resilience.

PHQ9 Depression: The PHQ-9 Depression Scale assessed depression severity. This instrument revealed comparable trends among its predictors. Age negatively correlated with depressive symptoms (coefficient of −0.02), suggesting older individuals experienced lower levels of depression. Gender differences remained, with females reporting significantly lower depressive symptoms than males (coefficient of −1.01). Educational attainment inversely correlated with depressive symptoms; individuals with higher education levels (high school graduate, some college credit, college degree) reported lower depression severity (coefficients of −1.35, −1.83, −2.55, respectively).

Insurance coverage significantly mitigated depressive symptoms; respondents with fully covered losses (coefficient of −1.93), mostly covered losses (coefficient of −1.56), or partially covered losses (coefficient of −1.0) reported lower depression levels. Higher income levels ($20,000–$30,000, $50,000–$75,000, $75,000–$100,000, and $100,000+) were linked to fewer depressive symptoms, emphasizing the protective role of financial stability on mental health.

Perceived Stress Scale (PSS): The Perceived Stress Scale (PSS) assessed respondents’ stress perception. This measure exhibited comparable significance patterns among its predictors. Age negatively correlated with perceived stress (coefficient = −0.01, *p* < 0.05), indicating that older respondents experienced lower stress levels. Education emerged as a significant factor, with individuals holding higher educational qualifications (high school diploma, some college credit, and college degree) reporting notably lower perceived stress levels (coefficients of −0.92, −1.45, −2.02, respectively).

Insurance coverage correlated with stress levels; respondents with fully covered losses had a coefficient of −1.1 (*p* < 0.001), while those with mostly covered losses had a coefficient of −0.8, indicating lower perceived stress. Household income levels ($20,000–$30,000, $30,000–$50,000, $50,000–$75,000, $75,000–$100,000, and $100,000+) were negatively correlated with stress, highlighting the importance of financial stability in lowering perceived stress levels.

### 5.3. Third Analysis: Regression Analysis of Psychological Parameters with Damage Levels

Another ordinary least squares regression analysis was conducted to examine the relationship between various socioeconomic and demographic factors and the psychological parameters experienced by respondents during Hurricane Katrina. However, in this analysis, levels of damage are included as independent variables along with the socio-demographics. The psychological parameters were categorized into four outcomes: “K6 Score,” “PTSD,” “PHQ9 Depression,” and “PSS.” Table 5 presents the regression results of factors associated with psychological outcomes integrating levels of damage as independent variables.

This analysis, presented in Table 5, builds upon the previous one by examining how socio-demographic factors, insurance coverage, and, crucially, the level of household physical damage collectively predict the mental health outcomes of the Displaced New Orleans Residents after Hurricane Katrina. The same psychological outcomes were measured: K6 Mental Illness Scale, PTSD Checklist, PHQ9 Depression, and Perceived Stress Scale. Here is a breakdown of the key findings for each psychological variable in this model:

K6 Mental Illness Scale: Significant predictors of psychological distress included age (coeff. = −0.03, *p* < 0.001), gender (coeff. = −0.47), white race (coeff. = −0.65), children in household (coeff. = 0.59, *p* < 0.05), education levels (coeff. = −1.46 to −2.7, *p* < 0.001), insurance coverage (all losses: coeff. = −1.64, *p* < 0.001; most losses: coeff. = −1.14), and higher household incomes ($50 k–$75 k and $100 k+ brackets). Physical damage also predicted distress, with no damage (coeff. = −1.66) and damaged/unlivable homes (coeff. = −0.73) associated with lower distress compared to destroyed homes, suggesting other factors beyond structural loss influence psychological outcomes.

PTSD Checklist: Key predictors included gender (coeff. = −1.89), white race (coeff. = −3.56, *p* < 0.001), children in household (coeff. = 1.47, *p* < 0.1), education levels (coeff. = −2.77 to −5.84), insurance coverage (all losses: coeff. = −5.58; most losses: coeff. = −4.28), and higher income levels across all brackets. Physical damage significantly predicted PTSD symptoms, with no damage (coeff. = −7.0), damaged but livable (coeff. = −2.49), and damaged/unlivable (coeff. = −2.44) homes all associated with lower symptoms relative to destroyed homes, indicating potential variations in coping mechanisms or support availability based on damage severity.

PHQ9 Depression: Significant predictors included age (coeff. = −0.02, *p* < 0.1), gender (coeff. = −0.98, *p* < 0.001), education levels (coeff. = −1.36 to −2.48, *p* < 0.001), insurance coverage (all losses: coeff. = −1.86; most losses: coeff. = −1.6; some losses: coeff. = −1.06, *p* < 0.05), and higher household incomes ($20 k–$30 k, $50 k–$75 k, $100 k+ brackets). Physical damage predicted depression, with no damage (coeff. = −2.25, *p* < 0.001), damaged but livable (coeff. = −0.84, *p* < 0.1), and damaged/unlivable (coeff. = −1.06, *p* < 0.001) homes associated with lower symptoms than destroyed homes, indicating potential influences from external aid, coping mechanisms, or support systems.

Perceived Stress Scale (PSS): Significant predictors included age (coeff. = −0.01, *p* < 0.05), education levels (coeff. = −0.92 to −2.01, *p* < 0.001), insurance coverage (all losses: coeff. = −1.11; most losses: coeff. = −0.83, *p* < 0.05), and higher household incomes across all brackets ($20 k–$30 k through $100 k+, *p* < 0.05). Unlike other psychological outcomes, physical damage showed non-significant effects across all categories, suggesting that factors beyond structural loss, such as coping mechanisms or social support, may be more influential in determining perceived stress levels.

## 6. Discussion

The regression analysis without considering physical damage highlights the significant role of various socioeconomic factors and insurance coverage in predicting psychological distress after Hurricane Katrina. Higher levels of education and better insurance coverage consistently emerged as protective factors associated with lower levels of general mental distress, PTSD symptoms, depression, and perceived stress. Conversely, households with children tended to report higher levels of distress and PTSD symptoms. Age also appeared to be a protective factor, with older individuals generally reporting lower distress. It is important to note that these relationships are observed without accounting for the extent of physical damage experienced by these individuals. The subsequent analysis, which includes physical damage as a predictor, will offer further insights into these complex relationships.

The inclusion of physical damage as a predictor in the second OLS regression analysis generally reduced the magnitude of the coefficients for some of the other predictors, such as age and having children, suggesting that some of the impact of these factors on mental health might be mediated through their association with the likelihood or severity of physical damage. However, higher education and better insurance coverage remained strong protective factors across all psychological outcomes, even after accounting for the level of physical damage. This highlights their independent and significant role in fostering psychological well-being after a disaster. The results for household income also remained largely consistent, suggesting that socioeconomic status plays a significant role in mental health recovery that is not solely explained by the extent of physical damage.

Physical damage emerged as a significant predictor for K6, PTSD, and depression, with individuals experiencing lower levels of housing damage generally reporting lower levels of psychological distress relative to those whose homes were destroyed. Respondents whose homes were not damaged or were damaged but still livable exhibited reduced distress levels across several psychological measures, while the destroyed category consistently showed the highest levels of distress. These findings suggest that the severity of housing loss following a disaster plays an important role in shaping psychological outcomes, with complete destruction associated with the greatest mental health burden.

Unlike the K6, PTSD, and PHQ-9 outcomes, perceived stress showed no statistically significant association with any level of physical damage. This divergence has a clear conceptual basis. The PCL and PHQ-9 assess trauma-specific and clinical depressive symptoms whose onset can be directly traced to the disaster event, whereas the PSS measures a global, present-oriented appraisal of how unpredictable and uncontrollable one’s current life circumstances feel [46]. By the time DNORS was administered in 2009–2010, four to five years had elapsed since Hurricane Katrina, and the severity of structural damage sustained in 2005 may have had diminishing direct relevance to respondents’ day-to-day perceived stress. Current socioeconomic conditions (income stability, insurance resolution, and housing security) were likely far more proximal determinants of perceived stress at the time of interview, which is consistent with the finding that income, education, and insurance remained significant PSS predictors even after damage was included in the model. These findings suggest that the PSS may be capturing the chronic stress of incomplete economic recovery rather than the acute psychological trauma of the disaster itself. Future research should formally test whether the pathway from physical damage to perceived stress operates primarily through socioeconomic resource depletion, and whether interactions between damage severity and resources such as insurance coverage and income further explain variation in long-term perceived stress outcomes.

In conclusion, this regression analysis, by incorporating physical damage as a key predictor, provides a more nuanced understanding of the factors influencing post-disaster mental health. It confirms the significant direct impact of physical damage while also underscoring the persistent and independent roles of socioeconomic factors like education, insurance coverage, and income in shaping psychological outcomes after a catastrophic event like Hurricane Katrina. These findings are crucial for policymakers in developing targeted interventions that address both the immediate physical needs and the long-term psychological well-being of disaster-affected communities. The comparison of the significance of each of the predictor variables for each of the outcome variables, i.e., building damage levels and psychological outcome variables are summarized in Table 6 and Table 7.

## 7. Limitations and Future Research Directions

While this study provides valuable insights into the psychological impact of disasters, several limitations should be acknowledged. The research depends on publicly accessible DNORS data files, which are constrained by the original data gathering methodologies, variable selection, and sample composition. The investigation was performed exclusively on the households that submitted comprehensive data for psychological evaluations. The omission of homes with incomplete data may result in selection bias, thereby causing an underestimating or overestimation of psychological distress in the impacted population. Future research may investigate the utilization of imputation methods or sensitivity analysis to enhance comprehension of the effects of absent data on the outcomes.

A significant disadvantage is the dependence on self-reported psychological assessments, such as the K6 mental illness scale, the post-traumatic stress disorder checklist (PCL), depression metrics, and the perceived stress scale (PSS). Self-report instruments, although prevalent in psychological research, are intrinsically vulnerable to recall bias, answer distortions, and social desirability effects. Participants may have either minimized or amplified their distress levels due to stigma or subjective interpretation of their symptoms. Future research should adopt a multimodal approach, including clinical assessments, structured interviews, and physiological stress indicators, to yield a more objective and thorough evaluation of psychological well-being.

This study utilized a cross-sectional research strategy, which restricts the capacity to deduce causal links between socio-demographic characteristics and psychological discomfort. A longitudinal study monitoring individuals over time would facilitate a comprehensive knowledge of recovery trajectories, resilience development, and long-term mental health consequences in communities affected by disasters.

A further limitation of this study is that analyses were conducted without applying DNORS sampling weights or adjusting variance estimates for the complex survey design. DNORS employed a multistage probability sampling strategy that oversampled certain subgroups, including residents of heavily damaged neighborhoods. As a result, unweighted estimates may disproportionately reflect the experiences of the most severely affected households, and standard errors may be modestly underestimated due to unaccounted clustering. While unweighted regression analysis is appropriate for the associative and exploratory aims of this study, readers should exercise caution in generalizing the findings to the full population of pre-Katrina Orleans Parish residents. Future research should apply the available DNORS weights and employ complex survey estimation procedures to produce population-representative estimates and more conservative tests of statistical significance.

## 8. Policy Implications

The findings of this study carry several concrete implications for disaster recovery policy and mental health intervention. First, insurance coverage emerged as the strongest and most consistent protective factor across all four psychological outcomes. This underscores the need for policies that expand access to affordable and comprehensive flood and property insurance, particularly in low-income and minority communities that are disproportionately underinsured. Policymakers should consider expanding federally subsidized insurance programs, such as the National Flood Insurance Program (NFIP), and coupling them with pre-disaster financial literacy and outreach campaigns to increase uptake among vulnerable households before disaster strikes.

Education was a robust protective factor across all psychological outcomes and at all levels of attainment, even after controlling for physical damage. This finding supports investment in post-disaster programs that provide immediate access to mental health resources through educational institutions, as well as longer-term educational support initiatives such as scholarship programs, vocational training, and adult education opportunities for displaced adults whose educational trajectories were disrupted by the disaster.

The consistently elevated psychological distress observed among households with children points to an urgent need for family-centered disaster mental health services. These should include school-based trauma counseling and psychological first aid programs deployed rapidly in the aftermath of a disaster, as well as sustained child and adolescent mental health services that extend well into the recovery period. Parental mental health support is equally important, as caregiver distress and child well-being are deeply interconnected in disaster contexts.

The pronounced racial disparities in both structural damage and psychological outcomes, with African American households bearing disproportionate harm across nearly all measures, call for equity-focused disaster recovery frameworks. Rather than allocating recovery resources based solely on property values or assessed damage costs, which tend to replicate pre-existing inequalities, recovery programs should direct resources proportionally to demonstrated need and pre-existing socioeconomic vulnerability. This includes targeted outreach to minority communities, culturally competent mental health services, and anti-displacement policies that prevent low-income residents from being permanently excluded from rebuilt neighborhoods.

The persistent association between income and psychological outcomes, even after accounting for damage severity, reinforces the importance of economic stabilization as a mental health intervention in its own right. Programs that provide direct financial assistance, employment support, and stable housing in the immediate aftermath of a disaster, such as FEMA’s Individuals and Households Program and HUD’s Community Development Block Grant Disaster Recovery funds, should be structured to prioritize the lowest-income households and be accompanied by mental health screening and referral services to address the psychological dimensions of economic loss.

## 9. Conclusions

This study enhances understanding of psychological impacts following disasters by analyzing data from the DNORS public files collected between 2009 and mid-2010. By examining the relationships between socio-demographic factors and psychological well-being—measured using the K6 scale, the PTSD checklist (PCL), depression indicators, and the perceived stress scale (PSS)—the research identifies at-risk populations that experienced elevated psychological distress. The regression analysis underscores the complex interplay of individual, social, and environmental factors shaping post-disaster mental health outcomes and highlights the need for targeted support for the most vulnerable communities. Addressing limitations such as reliance on self-reported data and cross-sectional design will enable more robust insights into community resilience and risk. Future research should incorporate longitudinal tracking, qualitative data, and multi-method approaches to inform evidence-based policies and interventions. This work lays a foundation for advancing disaster preparedness, mental health support systems, and resilience-building initiatives. By identifying key drivers of psychological distress, it informs strategies for aiding recovery, minimizing long-term harm, and strengthening the resilience of populations facing future crises.

## Figures and Tables

**Table 1:** Summary of Observations for Sociodemographic Variables (adapted from [37]).

Variables	Obs.	Variables	Obs.
**Level of damage**		**Household Income**	
No damage	73	Less than 20,000	424
Damaged, but someone could still live in it	479	$20,000–$29,999	232
Damaged so badly that someone couldn’t live in it	788	$30,000–$49,999	341
Destroyed	342	$50,000–$74,999	294
**Race**		$75,000–$99,999	128
African American or black	1054	Higher than $100,000	263
White	571	**Dwelling Type**	
Others (Latino, Asian, etc.)	57	One-family house	1194
**Gender**		Other types of dwelling ^1^	488
Female	1022	**Household Insurance**	
Male	660	All or almost all of my losses	184
**Age**		Most of my losses	259
18–95	1682	About half of my losses	186
**Employment Status**		Some of my losses	360
Employed	1046	Very few or none of my losses	244
Unemployed	636	No insurance	449
**Education Level**		**Homeownership**	
Less than high school	273	Homeowner	1217
High school graduate	366	Others	465
Some college credit	465	**Number of Children**	
College degree	578	Without children	417
**No. of HH members**	1682	With children	1265

^1^ Two-family house or duplex, apartment or project, mobile home, row house or townhouse, or other—please specify.

**Table 2:** Summary of Observations for Psychological Variables.

Variables	No. of Obs.	Mean	Standard Deviation	Min	Max
K6 Score: 0–24	1629	5.346839	4.95911	0	24
PTSD Checklist	1629	30.58134	14.04841	17	85
PHQ-9 Depression	1629	4.995703	5.819509	0	27
Perceived Stress Scale	1629	4.913444	3.428705	0	16

**Table 3:** Multinomial Logistic Regression of damage levels (adapted from [37]).

Independent Variables	Damage Levels					
	Not Damaged		Damaged but Someone Could Still Live in It		Damaged So Badly That Someone Couldn’t Live in It	
	RRR	Std. Err.	RRR	Std. Err.	RRR	Std. Err.
One-family house	0.56	0.36	0.70	0.20	0.72	0.18
Homeowner	0.33 *	0.42	0.89	0.24	1.00	0.20
Female	0.84	0.29	1.09	0.16	1.26	0.14
White population	3.93 *	0.37	4.58 *	0.21	1.01	0.19
Other races	2.78	0.89	4.55 *	0.48	1.94	0.46
Households with children	0.53	0.35	0.65 *	0.21	0.64 *	0.19
High school graduate	1.67	0.53	0.71	0.26	0.96	0.22
Some college credit	0.76	0.57	0.68	0.25	0.93	0.21
College degree	2.40	0.57	1.17	0.28	1.18	0.25
Employed (2005)	0.73	0.32	0.66 *	0.17	0.79	0.15
Household Insurance Status						
All losses covered	3.44 *	0.56	4.81 *	0.38	2.48 *	0.36
Most losses covered	0.54	0.57	1.84 *	0.30	1.22	0.26
Half losses covered	0.19 *	0.84	0.84	0.32	1.24	0.27
Some losses covered	0.39	0.56	1.09	0.26	1.09	0.22
Few losses covered	1.01	0.43	0.79	0.27	0.81	0.22
Age	1.03 *	0.01	1.00	0.006	1.01 *	0.005
Number of household members	0.72 *	0.14	0.99	0.05	1.01	0.04
Household income levels						
20 k–30 k	0.77	0.49	0.67	0.26	0.76	0.22
30 k–50 k	0.65	0.50	0.67	0.25	0.98	0.21
50 k to 75 k	1.00	0.51	0.67	0.27	0.91	0.23
75 k to 100 k	0.67	0.66	0.28 *	0.36	0.82	0.29
100 k+	2.78	0.55	0.93	0.33	1.22	0.30
Constant	0.27	0.88	2.10	0.45	1.91	0.39
	Destroyed (base outcome)					

Note: RRR = Relative Risk Ratio; * = the significance of the RRRs and coefficients of each of the independent variables is measured at *p* < 0.05.

**Table 4:** Ordinary Least Squares Regression of psychological variables without physical damage as a predictor.

Independent Variables	Psychological Parameters							
	K6 Scale		PTSD		PHQ9 Depression		PSS	
	Coeff.	Std. Err.	Coeff.	Std. Err.	Coeff.	Std. Err.	Coeff.	Std. Err.
One-family house	−0.18	0.30	−0.69	0.84	−0.24	0.36	−0.2	0.21
Homeowner	−0.16	0.36	0.63	1	0.39	0.43	0.44 +	0.25
Age	−0.04 ***	0.01	−0.02	0.02	−0.02 *	0.01	−0.01 *	0.01
Female	−0.50 *	0.24	−1.96 ***	0.67	−1.01 ***	0.29	−0.22	0.17
White population	−0.67 *	0.30	−4.07 ***	0.83	−0.51	0.36	−0.32	0.21
Other races	0.54	0.66	0.45	1.85	0.3	0.79	0.49	0.46
Households with children	0.66 *	0.29	1.74 *	0.81	0.49	0.35	0.12	0.2
High school graduate	−1.46 ***	0.40	−2.74 *	1.11	−1.35 ***	0.48	−0.92 ***	0.28
Some college credit	−2.06 ***	0.39	−4.6 ***	1.09	−1.83 ***	0.47	−1.45 ***	0.27
College degree	−2.75 ***	0.43	−6.08 ***	1.19	−2.55 ***	0.51	−2.02 ***	0.3
Employed (2005)	0.04	0.26	−0.1	0.71	−0.27	0.31	0	0.18
Household Insurance Status								
All losses covered	−1.66 ***	0.48	−5.82 *	1.34	−1.93 ***	0.57	−1.1 ***	0.34
Most losses covered	−1.08 *	0.44	−4.12 *	1.23	−1.56 ***	0.53	−0.8 *	0.31
Half losses covered	−0.14	0.48	−2.02	1.33	−0.57	0.57	−0.33	0.33
Some losses covered	−0.70 +	0.40	−1.82	1.1	−1 *	0.47	−0.42	0.28
Few losses covered	−0.51	0.41	−0.7	1.13	−0.25	0.49	0.03	0.28
Household income levels								
20 k–30 k	−0.72 +	0.40	−2.58 *	1.13	−1.08 *	0.48	−0.9 ***	0.28
30 k–50 k	−0.61	0.38	−3.42 ***	1.05	−0.76 +	0.45	−0.58 *	0.26
50 k to 75 k	−1.09 *	0.41	−4.59 ***	1.15	−1.52 ***	0.49	−0.99 ***	0.29
75 k to 100 k	−1.09 *	0.53	−4.2 ***	1.48	−1.63 *	0.63	−0.89 *	0.37
100 k+	−1.08 *	0.47	−3.86 ***	1.32	−1.25 *	0.57	−0.92 *	0.33
Constant	10.55	0.57	41.15	1.58	9.82	0.68	7.91	0.4

Note: + *p* < 0.1, * *p* < 0.05, *** *p* < 0.001.

**Table 5:** Ordinary Least Squares Regression of psychological variables with physical damage as predictor.

Independent Variables	Psychological Parameters							
	K6 Scale		PTSD		PHQ9 Depression		PSS	
	Coeff.	Std. Err.	Coeff.	Std. Err.	Coeff.	Std. Err.	Coeff.	Std. Err.
One-family house	−0.22	0.3	−0.87	0.84	−0.31	0.36	−0.21	0.21
Homeowner	−0.21	0.36	0.35	0.99	0.31	0.43	0.43 +	0.25
Age	−0.03 ***	0.01	−0.01	0.02	−0.02 +	0.01	−0.01 *	0.01
Female	−0.47 +	0.24	−1.89 ***	0.67	−0.98 ***	0.29	−0.21	0.17
White population	−0.65 *	0.31	−3.56 ***	0.86	−0.4	0.37	−0.31	0.22
Other races	0.63	0.67	1.06	1.85	0.49	0.8	0.5	0.47
Households with children	0.59 *	0.29	1.47 +	0.81	0.39	0.35	0.1	0.2
High school graduate	−1.46 ***	0.4	−2.77 *	1.11	−1.36 ***	0.48	−0.92 ***	0.28
Some college credit	−2.06 ***	0.39	−4.67 ***	1.08	−1.85 ***	0.47	−1.44 ***	0.27
College degree	−2.7 ***	0.43	−5.84 ***	1.18	−2.48 ***	0.51	−2.01 ***	0.3
Employed (2005)	0.03	0.26	−0.2	0.71	−0.31	0.3	0	0.18
Household Insurance Status								
All losses covered	−1.64 ***	0.48	−5.58 ***	1.34	−1.86 ***	0.58	−1.11 ***	0.34
Most losses covered	−1.14 *	0.44	−4.28 ***	1.23	−1.6 ***	0.53	−0.83 *	0.31
Half losses covered	−0.2	0.48	−2.37 +	1.33	−0.64	0.57	−0.35	0.34
Some losses covered	−0.75 +	0.4	−2.06 +	1.1	−1.06 *	0.47	−0.44	0.28
Few losses covered	−0.55	0.41	−0.85	1.13	−0.29	0.48	0.02	0.28
Household income levels								
20 k–30 k	−0.74 +	0.4	−2.65 *	1.12	−1.11 *	0.48	−0.9 ***	0.28
30 k–50 k	−0.59	0.38	−3.38 ***	1.04	−0.74	0.45	−0.57 *	0.26
50 k to 75 k	−1.08 *	0.41	−4.6 ***	1.15	−1.52 ***	0.49	−0.98 ***	0.29
75 k to 100 k	−1.06 +	0.53	−4.32 ***	1.47	−1.65 *	0.63	−0.87 *	0.37
100 k+	−1.02 *	0.47	−3.65 *	1.31	−1.17 *	0.57	−0.9 *	0.33
Household damage levels								
Not Damaged	−1.66 *	0.59	−7 ***	1.65	−2.25 ***	0.71	−0.46	0.42
Damaged but livable	−0.38	0.36	−2.49 *	1	−0.84 +	0.43	−0.04	0.25
Damaged and unlivable	−0.73 *	0.31	−2.44 ***	0.86	−1.06 ***	0.37	−0.14	0.22
Constant	11.03	0.61	43.22	1.7	10.61	0.73	7.99	0.43

Note: + *p* < 0.1, * *p* < 0.05, *** *p* < 0.001.

**Table 6:** Independent Variables Predicting Levels of Damage and Psychological Outcomes (Without Damage Levels as an Independent Variable).

Independent Variables	Outcome Variables				
	Damage Levels	K6 Scale	PTSD	Depression	PSS
Older adult	More likely non-destroyed	Lower	NS	Lower	Lower
Homeowner	Less likely non-destroyed	NS	NS	NS	Higher
White population	More likely non-destroyed	Lower	Lower	NS	NS
Households with children	Less likely non-destroyed	Higher	Higher	NS	NS
Higher income	Mostly NS	Lower	Lower	Lower	Lower
Insured	More likely non-destroyed	Lower	Lower	Lower	Lower
Higher education	NS	Lower	Lower	Lower	Lower
Female	NS	Lower	Lower	Lower	NS
Employed (2005)	Less likely livable vs. destroyed	NS	NS	NS	NS

**Table 7:** Independent Variables Predicting Levels of Damage and Psychological Outcomes (With Damage Levels as an Independent Variable).

Independent Variables	Outcome Variables				
	Damage Levels	K6 Scale	PTSD	Depression	PSS
Older adult	More likely non-destroyed	Lower	NS	Lower	Lower
Homeowner	Less likely non-destroyed	NS	NS	NS	Higher
White population	More likely non-destroyed	Lower	Lower	NS	NS
Households with children	Less likely non-destroyed	Higher	Higher	NS	NS
Higher income	Mostly NS	Lower	Lower	Lower	Lower
Insured	More likely non-destroyed	Lower	Lower	Lower	Lower
Higher education	NS	Lower	Lower	Lower	Lower
Female	NS	Lower	Lower	Lower	NS
Employed (2005)	Less likely livable vs. destroyed	NS	NS	NS	NS
Damage: Not damaged	—	Lower	Lower	Lower	NS
Damage: Livable	—	NS	Lower	Lower	NS
Damage: Unlivable	—	Lower	Lower	Lower	NS

Note: NS = Not Significant.

## Data Availability

The data presented in this study are openly available at https://www.rand.org/education-employment-infrastructure/projects/dnors/data/public.html (accessed on 30 December 2025).
